# Case report: Successful use of mepolizumab for immune checkpoint inhibitors–induced hypereosinophilic syndrome in two patients with solid malignancies

**DOI:** 10.3389/fonc.2023.1079034

**Published:** 2023-01-26

**Authors:** Chiara Lazzari, Mona Rita Yacoub, Corrado Campochiaro, Alessandra Bulotta, Diego Palumbo, Francesca Rita Ogliari, Lorenzo Dagna, Silvia Marchesi, Maurilio Ponzoni, Vanesa Gregorc

**Affiliations:** ^1^ Department of Oncology, Candiolo Cancer Institute Fondazione Piemontese per l'Oncologia-Istituto di Ricerca a Carattere Scientifico (FPO-IRCCS) Candiolo, Torino, Italy; ^2^ Department of Rheumatology and Clinical Immunology, Istituto di Ricerca a Carattere Scientifico (IRCCS) San Raffaele Hospital, Milano, Italy; ^3^ Department of Oncology, Istituto di Ricerca a Carattere Scientifico (IRCCS) San Raffaele Hospital, Milano, Italy; ^4^ Department of Radiology, Istituto di Ricerca a Carattere Scientifico (IRCCS) San Raffaele Hospital, Milano, Italy; ^5^ Vita-Salute San Raffaele University, Milano, Italy; ^6^ Pathology Unit, Istituto di Ricerca a Carattere Scientifico (IRCCS) San Raffaele Scientific Institute, Milano, Italy

**Keywords:** HES, NSCLC, mepolizumab, immune checkpoint inhibitor, mesothelioma

## Abstract

Hypereosinophilic syndrome (HES) represents a group of blood disorders characterized by an absolute eosinophil count (AEC) > 1.5 × 103/μl in the peripheral blood, which eventually extravasate and cause organ damage. It can be primary or secondary to infections or tumors. The infiltration of eosinophils in tissue and organs is associated with different disorders and, in some cases, with life-threatening manifestations. Albeit the pathogenesis of HES in patients with solid tumo\rs is not yet clarified; recently, HES has also been described as an immune-related adverse event in patients with solid tumors receiving immune checkpoint inhibitors. Treatment of HES is still debated, especially in patients with concomitant solid tumors, and different drugs including imatinib, hydroxyurea, interferon-ɑ, glucocorticoids, and the monoclonal antibody targeting circulating IL-5 mepolizumab have been proposed according to the underlying cause and the severity of HES. Herein, we describe, for the first time, the successful use of mepolizumab for the treatment of immune checkpoint–induced HES in two patients with metastatic solid tumor.

## Introduction

Hypereosinophilic syndrome (HES) represents a group of blood disorders, which are characterized by an absolute eosinophil count (AEC) > 1.5 × 10^3^/μl with evidence of eosinophil-related clinical manifestations. HES can be either (1) *primary* in the setting of a clonal myeloid or eosinophilic neoplasm (2); *secondary* (reactive) when eosinophilic expansion is polyclonal, due to parasitic infections, solid tumors, and T cell lymphoma; or (3) *idiopathic* when the underlying cause remains unknown despite careful evaluation and the exclusion of specific syndromes associated with hypereosinophilia, such as eosinophilic granulomatosis with polyangitis and certain immunodeficiencies (1). Eosinophil production, survival, and chemotaxis are controlled by different cytokines, the most important being interleukin (IL)-5. Additionally, eosinophils can amplify inflammatory processes, recruiting more cells. Different organs can be affected. The most commonly affected ones include skin, lungs, heart, and central nervous system. Paraneoplastic HES in patients with solid tumors has also been already reported.

We, herein, report two patients affected by solid tumors who developed HES during the treatment with immune check point inhibitor and who were successfully treated with the anti–IL-5 monoclonal antibody mepolizumab. We also provide an overview of the literature focusing on the clinical data associated with HES and the therapeutic strategies currently used for treating this condition.

## Case details 1

**Figure 1:**
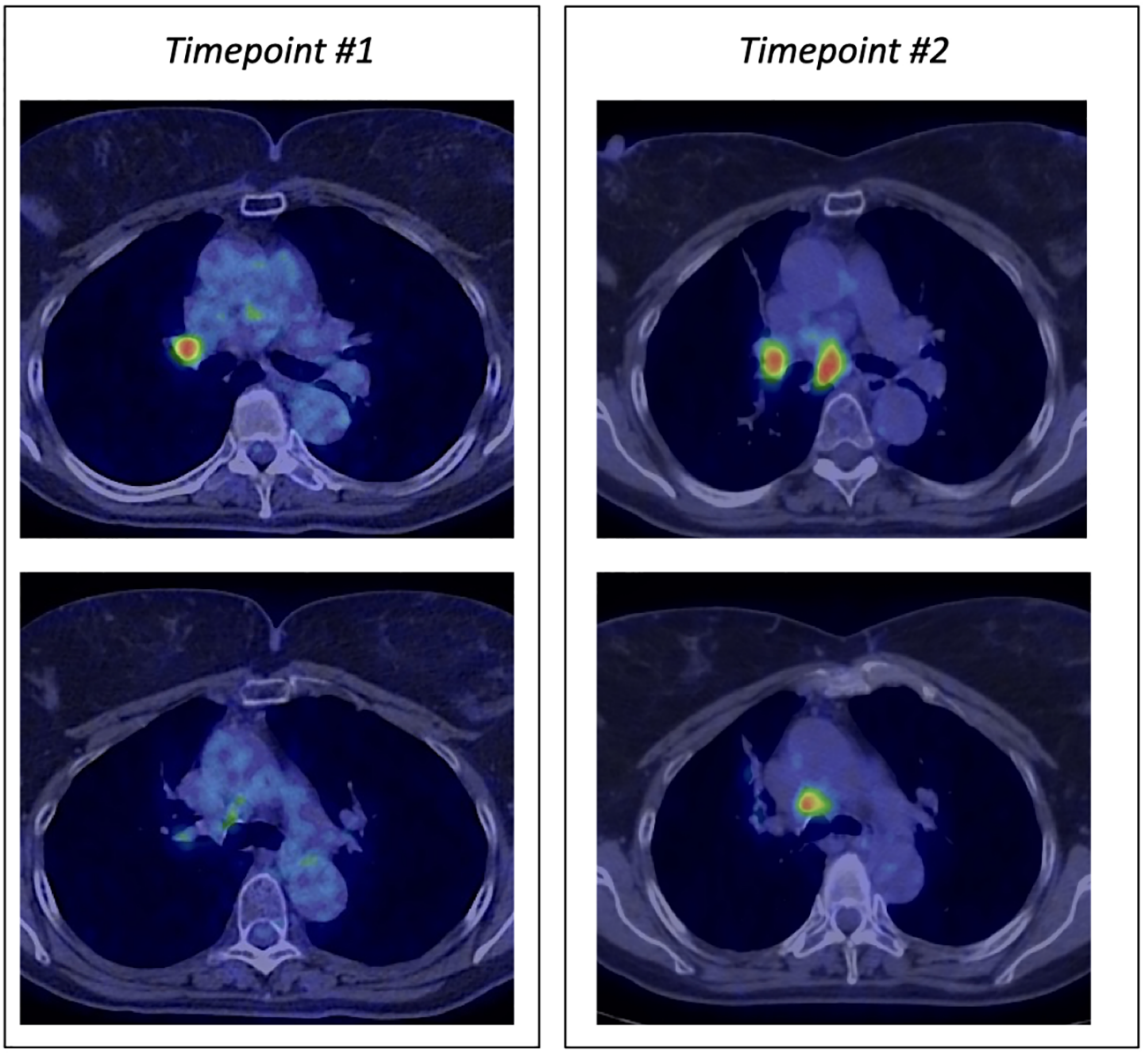
PET scan at baseline showing the progression at mediastinal lymph nodes.

**Figure 2:**
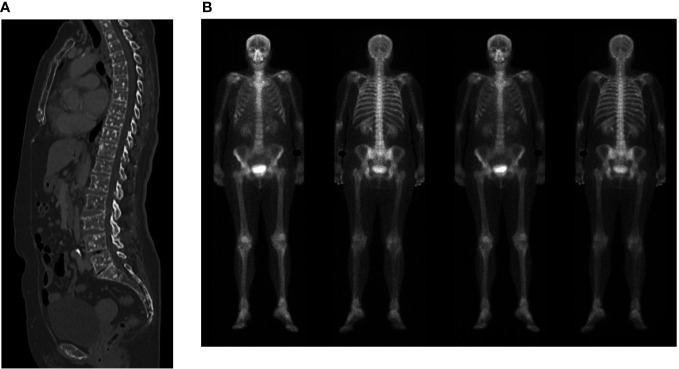
**(A)** Computed tomography scan showing multiple bone lesions. **(B)** Bone scan showing no evidence of bone metastases.

**Figure 3:**
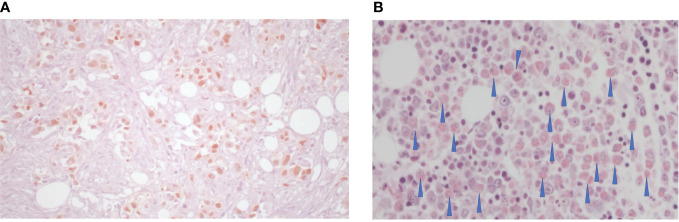
**(A)** Hematoxylin and eosin staining of adenocarcinoma of the lung, TTF1+ associated with fibrosis (70%); **(B)** Giemsa eosinophilic infiltrate, as indicated by the arrows.

A 69-year-old former smoker patient was diagnosed with stage IV lung adenocarcinoma with pleural lesions in 2016. Molecular analysis revealed the presence of a mutation in the KRAS gene (Q61H), associated with immunoreactivity for PD-L1 in more than 90% of tumor population cells. Based on tumor’s molecular characteristics, therapy with pembrolizumab was started with a partial response lasting for 28 months, when tumor progressed on mediastinal lymph nodes (**Figure 1**). To differentiate between nodal immune flare related to pembrolizumab and tumor progression, the patient underwent bronchial ultrasound transbronchial fine needle aspiration. Cytological analysis identified the presence of cancer cells, and chemotherapy with pemetrexed was added to pembrolizumab. The computed tomography (CT) scan performed 2 months following the beginning of therapy showed a partial response on lymph nodes with the appearance of multiple bone lesions, as reported in **Figure 2A**, not revealed by the bone scan (**Figure 2B**). Due to the inconsistency between the CT scan and the bone scan, to confirm or exclude the presence of bone metastases, the patient underwent a bone marrow biopsy (BMB), which identified a small metastatic deposit of adenocarcinoma consistent, by virtue of immunophenotypical features (TTF-1+, CK+), with origin from the lung. Because the patient was not symptomatic, she refused to start treatment with docetaxel and was maintained on pembrolizumab in combination with pemetrexed. After 10 months, routine blood tests revealed thrombocytopenia (39,000/μl) associated with hypereosinophilia (7.6 × 10^9^/L). Two days after patient developed dysarthria with a leftward deviation of the lip. A brain magnetic resonance imaging (MRI) showed the presence of ischemia in the right fronto-insular cortex extending to the ipsilateral semioval center and corona radiata. No stenosis of the craniocervical arteries at the angio-CT scan or cardiac abnormalities at the echocardiography were identified. Based on these findings, we concluded for a cerebral stroke in HES. To better define the cause of thrombocytopenia, a second BMB was performed. At BMB, a much prominent metastasis of adenocarcinoma (with the same histopathological and immunophenotypical features), embedded within prominent fibrosis (70% of biopsy volume) and eosinophils (**Figures 3A, B**) was disclosed. Since the thorax abdomen CT scan did not show disease progression, we suspected an immune checkpoint inhibitor–induced HES. Unfortunately, patient’s conditions rapidly worsened with further platelet decrease, raise of inflammatory markers (CRP-170 mg/L, with normal < 6 mg/L; procalcitonin 36 ng/ml, with normal range < 0,05 ng/ml), and the development of sepsis. Prednisone at the dose of 25 mg was started. Despite the reduction in the eosinophil count, no clear improvement of patient’s conditions or platelet increase was observed. The IL-5 inhibitor mepolizumab at the dose of 100-mg subcutaneous was then started after written-informed consent. At that time, platelet level was 33,000/μl. Ten days after, the value increased to 57,000/μl, and then to 156,000/μl 1 month later. Repeated blood tests showed complete resolution of eosinophilia, associated with a significant clinical improvement. No additional dose of mepolizumab was administered.

## Case details 2

**Figure 4:**
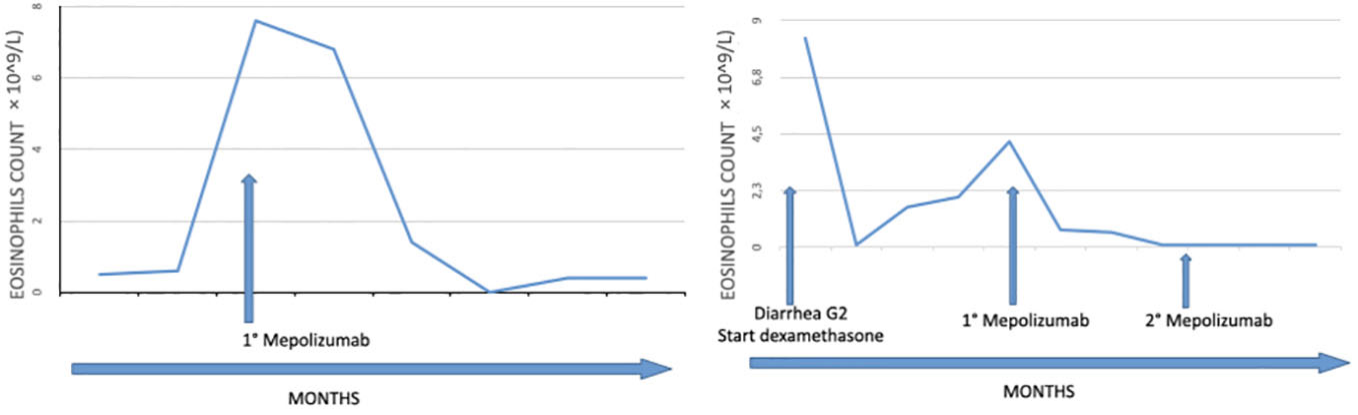
Timeline showing the clinical history of both patients.

A 64-year-old man was diagnosed with malignant pleural mesothelioma in November 2019. In his medical history, he had a diagnosis of mycosis fungoides in 2006 with no active treatment. Following first-line chemotherapy, including carboplatin pemetrexed, he underwent disease progression in September 2020 and a second-line treatment with nivolumab plus ipilimumab was started. Therapy was well tolerated with no significant adverse event. The positron emission tomography (PET) scan showed a partial response. Since July 2021, he developed hypereosinophilia (up to 8 × 10^9^/L), as shown in **Figure 4**. Moreover, the worsening of mycosis fungoides and the onset of diarrhea G2 according to common terminology criteria for adverse events (CTCAE) 4.0 were observed. Immunotherapy was temporary discontinued, and prednisone at the dose of 25-mg daily was started with rapid decrease in peripheral eosinophil count. At the time of immune checkpoint re-challenge, the eosinophil count increased back to 4.2 × 10^9^/L, with concomitant elevation in the level of serum IgE (over 1500 IU/ml) and serum tryptase (16 ug/L) and diarrhea worsening. Based on these findings, we diagnosed immune checkpoint inhibitor–induced HES with colitis. Mepolizumab at the dose of 300 mg was then started. Subsequent laboratory tests showed a rapid decrease in the eosinophilic count, reaching 1 × 10^9^/L 2 weeks later. Immunotherapy was continued with no further adverse events until tumor progression.

## Discussion

HES is a rare disorder, difficult to diagnose, and characterized by heterogeneous manifestations, including clinical and hematological characteristics of myeloproliferative disease, such as splenomegaly, anemia, and myelofibrosis.

Eosinophils are produced by the bone marrow under the stimulation of IL-3, IL-5, and granulocyte/macrophage colony stimulating factor (GM-CSF; 2). IL-5 is responsible for eosinophils terminal differentiation, activation, and survival. Once eosinophils migrate into tissues (3), they cause damage by generating oxidative stress through eosinophil peroxidase (EPO), cytotoxic proteins, including eosinophil cationic protein (ECP), eosinophil-derived neurotoxin (EDN), or through the secretion of proteolytic enzymes. Eosinophils release different cytokines, namely, IL-10, IL-14, IL-9, IL-13, IL-25, IL-12, interferon (IFN)-γ, tumor necrosis factor (TNF)-α, IL-1β, IL-6, and IL-8, which are responsible for maintaining homeostasis and immune regulation (4–7). As a consequence, the accumulation of eosinophils in tissue might favor the development of thrombosis, fibrosis, angiogenesis, tissue remodeling, platelet, and endothelial cell activation (8–10).

Few data are available regarding HES in patients with solid tumors, and the diagnosis of paraneoplastic HES is often one of exclusion. HES has been observed in many solid tumor types, namely, lung cancer, renal cell carcinoma, cervical cancer, gastrointestinal cancer, testicular, and breast cancer. The pathogenesis of HES in the setting of solid tumors is the subject of current research (11–13). Data from literature showed a favorable prognostic role for eosinophilic infiltration in some tumor types, namely, gastric cancer (14) and colorectal tumor (15), and a correlation with poor prognosis (16), or the presence of tumor recurrence (17) in other tumors.

Generally, for patients with mild eosinophilia (0.5–1.5 × 109/L) and without organ dysfunction, a watch-and-wait approach is suggested. Glucocorticoids are used in those with idiopathic HES. Alternatively, high dose of mepolizumab (300 mg every 4 weeks), a monoclonal antibody targeting circulating IL-5, has proven to be effective and well tolerated (18), thus allowing glucocorticoid sparing effects in patients with frequent flare (19). Nonetheless, no data are available in the subset of patients developing HES secondary to immune checkpoint inhibitors.

The association of HES and immunotherapy is another aspect worth investigating. To the best of our knowledge, few cases of HES induced by immune checkpoint inhibitors have been published in the English literature (20, 21). In one case, asymptomatic HES was noted during therapy but improved following the temporary suspension of treatment. Eosinophilia recurred following the continuation of therapy (22). Another patient developed eosinophilic pneumonia, whereas eosinophilic oesophagitis was described in a third patients. In all the cases, a successfully treatment with medium doses of steroids (prednisone at the dose of 25 mg) was administered. Conversely, in one of our patients, HES was associated with life-threatening manifestations, and an unexpected deterioration of the clinical condition despite steroids, thus prompting us to use the anti–IL-5 therapy mepolizumab. Due to the poor status of the patient and the sepsis, we did not use the standard dose of 300 mg of mepolizumab but a reduced dose of 100 mg.

To the best of our knowledge, the cases here reported represent the first evidence of the successful use of mepolizumb in patients developing HES under immune checkpoint inhibitors. **Figure 4** summarizes the clinical history of both patients and the levels of eosinophils before and following treatment with mepolizumab.

## Conclusions

HES is a rare disorder that may occur as a paraneoplastic syndrome. Recently, it has been described as an adverse event in the course of treatment with immune checkpoint inhibitors. Steroids do not represent the only option for the treatment of immune checkpoint-related adverse events, especially in the case of steroid refractory or relapsing adverse events (23, 24), as we have described in our patients. Mepolizumab was effective for the management of immune checkpoint inhibitor-related HES.

## Data availability statement

The raw data supporting the conclusions of this article will be made available by the authors, without undue reservation.

## Ethics statement

The studies involving human participants were reviewed and approved by the ethic committee from IRCCS San Raffaele Hospital. The patients/participants provided their written informed consent to participate in this study. Written informed consent was obtained from the individual(s) for the publication of any potentially identifiable images or data included in this article.

## Author contributions

CL, MY, CC, VG contributed to the conception of the study and helped perform the analysis with constructive discussions. VG, CL, FO contributed to manage and treat the patient. MP were responsible for histopathological evaluation. CL, MY, CC, VG, FO wrote the manuscript. All authors contributed to the article and approved the submitted version.

## References

[1] Curtis C Ogbogu P. Hypereosinophilic syndrome. Clin Rev Allergy Immunol (2016) 2:240–51. doi: 10.1007/s12016-015-8506-7 26475367

[2] Leru PM. Eosinophilic disorders: Evaluation of current classification and diagnostic criteria, proposal of a practical diagnostic algorithm. Clin Transl Allergy (2019) 9:36. doi: 10.1186/s13601-019-0277-4 31367340 PMC6657042

[3] Leiferman KM Peters MS. Eosinophil-related disease and the skin. J Allergy Clin Immunol Pract (2018) 5:1462–1482.e6. doi: 10.1016/j.jaip.2018.06.002 29902530

[4] Fulkerson PC Rothenberg ME. Eosinophil development, disease involvement, and therapeutic suppression. Adv Immunol (2018) 138:1–34. doi: 10.1016/bs.ai.2018.03.001 29731004

[5] Liao W Long H Chang CC Lu Q. The eosinophil in health and disease: From bench to bedside and back. Clin Rev Allergy Immunol (2016) 2:125–39. doi: 10.1007/s12016-015-8507-6 26410377

[6] Rosenberg HF Dyer KD Foster PS. Eosinophils: changing perspectives in health and disease. Nat Rev Immunol (2013) 13:9–22. doi: 10.1038/nri3341 23154224 PMC4357492

[7] Blanchard C Rothenberg ME. Biology of the eosinophil. Adv Immunol (2009) 101:81–121. doi: 10.1016/S0065-2776(08)01003-1 19231593 PMC4109275

[8] Varricchi G Galdiero MR Loffredo S Lucarini V Marone G Mattei F. Eosinophils: The unsung heroes in cancer? Oncoimmunology (2017) 2:e1393134. doi: 10.1080/2162402X.2017.1393134 PMC5749653 29308325

[9] Amini-Vaughan ZJ Martinez-Moczygemba M Huston DP. Therapeutic strategies for harnessing human eosinophils in allergic inflammation, hypereosinophilic disorders, and cancer. Curr Allergy Asthma Rep (2012) 5:402–12. doi: 10.1007/s11882-012-0290-3 PMC3729434 22875242

[10] De Giacomi F Decker PA Vassallo R Ryu JH. Acute eosinophilic pneumonia. correlation of clinical characteristics with underlying cause. Chest (2017) 152(2):379–85. doi: 10.1016/j.chest.2017.03.001 28286263

[11] Pandit R Scholnik A Wulfekuhler L Dimitrov N. Non-small-cell lung cancer associated with excessive eosinophilia and secretion of interleukin-5 as a paraneoplastic syndrome. Am J Hematol (2007) 3:234–7. doi: 10.1002/ajh.20789 17160990

[12] Kanaji N Watanabe N Kita N Bandoh S Tadokoro A Ishii T. Paraneoplastic syndromes associated with lung cancer. World J Clin Oncol (2014) 3:197–223. doi: 10.5306/wjco.v5.i3.197 PMC4127595 25114839

[13] De Giacomi F Vassallo R Yi ES Ryu JH. Acute eosinophilic pneumonia. causes, diagnosis, and management. Respir Crit Care Med (2018) 197(6):728–36. doi: 10.1164/rccm.201710-1967CI 29206477

[14] Iwasaki K Torisu M Fujimura T. Malignant tumor and eosinophils. i. prognostic significance in gastric cancer. Cancer (1986) 6:1321–7. doi: 10.1002/1097-0142(19860915)58:6<1321::AID-CNCR2820580623>3.0.CO;2-O 3742457

[15] Fernández-Aceñero MJ Galindo-Gallego M Sanz J Aljama A. Prognostic influence of tumor-associated eosinophilic infiltrate in colorectal carcinoma. Cancer. (2000) 7:1544–8. doi: 10.1002/(SICI)1097-0142(20000401)88:7<1544::AID-CNCR7>3.0.CO;2-S 10738211

[16] El-Osta H El-Haddad P Nabbout N. Lung carcinoma associated with excessive eosinophilia. J Clin Oncol (2008) 20:3456–7. doi: 10.1200/JCO.2007.15.8899 18612162

[17] Lo CH Jen YM Tsai WC Chung PY Kao WY. Rapidly evolving asymptomatic eosinophilia in a patient with lung adenocarcinoma causes cognitive disturbance and respiratory insufficiency: Case report. Oncol Lett (2013) 2:495–8. doi: 10.3892/ol.2012.1020 PMC3573146 23420572

[18] Roufosse F Kahn JE Rothenberg ME Wardlaw AJ Klion AD Kirby SY. Efficacy and safety of mepolizumab in hypereosinophilic syndrome: A phase III, randomized, placebo-controlled trial. J Clin Immunol (2020) 6:1397–405. doi: 10.1016/j.jaci.2020.08.037 PMC9579892 32956756

[19] Kuang FL Fay MP Ware JA Wetzler L Holland-Thomas N Brown T. Long-term clinical outcomes of high-dose mepolizumab treatment for hypereosinophilic syndrome. J Allergy Clin Immunol Pract (2018) 5:1518–1527.e5. doi: 10.1016/j.jaip.2018.04.033 PMC6173586 29751154

[20] Hattori Y Matsuyama K Shu E Seishima M. Eosinophilic pneumonia and esophagitis in a patient with malignant melanoma treated with nivolumab. J Dermatol (2019) 12:e454–5. doi: 10.1111/1346-8138.15030 31353501

[21] Jodai T Yoshida C Sato R Kakiuchi Y Sato N Iyama S. A potential mechanism of the onset of acute eosinophilic pneumonia triggered by an anti-PD-1 immune checkpoint antibody in a lung cancer patient. Immun Inflammation Dis (2019) 1:3–6. doi: 10.1002/iid3.238 PMC6416763 30461210

[22] Lou Y Marin-Acevedo JA Vishnu P Manochakian R Dholaria B Soyano A. Hypereosinophilia in a patient with metastatic non-small-cell lung cancer treated with antiprogrammed cell death 1 (anti-PD-1) therapy. Immunotherapy. (2019) 7:577–84. doi: 10.2217/imt-2018-0128 30943864

[23] Campochiaro C Farina N Tomelleri A Ferrara R Lazzari C De Luca G. Tocilizumab for the treatment of immune-related adverse events: a systematic literature review and a multicentre case series. Eur J Intern Med (2021) 93:87–94. doi: 10.1016/j.ejim.2021.07.016 34391591

[24] Campochiaro C Farina N Tomelleri A Ferrara R Viola S Lazzari C. Dagna l autoantibody positivity predicts severity of rheumatic immune-related adverse events to immune-checkpoint inhibitors. Eur J Intern Med (2022) 103:95–9. doi: 10.1016/j.ejim.2022.07.005 35821192

